# Metabolism and functions of docosahexaenoic acid‐containing membrane glycerophospholipids

**DOI:** 10.1002/1873-3468.12825

**Published:** 2017-09-07

**Authors:** Daisuke Hishikawa, William J. Valentine, Yoshiko Iizuka‐Hishikawa, Hideo Shindou, Takao Shimizu

**Affiliations:** ^1^ Department of Lipid Signaling National Center for Global Health and Medicine Shinjuku‐ku Tokyo Japan; ^2^ Department of Lipid Science The University of Tokyo Bunkyo‐ku Japan; ^3^ AMED Chiyoda‐ku Tokyo Japan; ^4^ Department of Lipidomics Graduate School of Medicine The University of Tokyo Bunkyo‐ku Japan

**Keywords:** lysophospholipid acyltransferase, membrane remodeling, omega‐3 fatty acid

## Abstract

Omega‐3 (ω‐3) fatty acids (FAs) such as docosahexaenoic acid (DHA) and eicosapentaenoic acid (EPA) are known to have important roles in human health and disease. Besides being utilized as fuel, ω‐3 FAs have specific functions based on their structural characteristics. These functions include serving as ligands for several receptors, precursors of lipid mediators, and components of membrane glycerophospholipids (GPLs). Since ω‐3 FAs (especially DHA) are highly flexible, the levels of DHA in GPLs may affect membrane biophysical properties such as fluidity, flexibility, and thickness. Here, we summarize some of the cellular mechanisms for incorporating DHA into membrane GPLs and propose biological effects and functions of DHA‐containing membranes of several cell and tissue types.

## Abbreviations

**AA**, arachidonic acid

**ACS**, acyl‐CoA synthetase

**AdipoR1**, adiponectin receptor 1

**ADP**, adenosine diphosphate

**ALA**, alpha‐linolenic acid

**AMPK**, adenosine monophosphate‐activated protein kinase

**Aβ**, amyloid‐beta

**BBB**, blood–brain barrier

**BRB**, blood–retinal barrier

**DHA**, docosahexaenoic fatty acid

**ELOVL**, elongation of very long‐chain fatty acid

**EPA**, eicosapentaenoic acid

**FA**, fatty acid

**FABP**, fatty acid‐binding protein

**FADS**, fatty acid desaturase

**GPL**, glycerophospholipid

**LCFA**, long‐chain fatty acid

**LPAAT**, lysophosphatidic acid acyltransferase

**LPC**, lysophosphatidylcholine

**LPLAT**, lysophospholipid acyltransferase

**MCFA**, medium chain fatty acid

**Mfsd2a**, major facilitator domain‐containing protein 2a

**MUFA**, monounsaturated fatty acid

**OS**, outer segment

**PA**, phosphatidic acid

**PC**, phosphatidylcholine

**PE**, phosphatidylethanolamine

**PGC1α**, peroxisome proliferator‐activated receptor gamma coactivator‐1 alpha

**PLA**, phospholipase

**PPAR**, peroxisome proliferator‐activated receptor

**PUFA**, polyunsaturated fatty acid

**RPE**, retinal pigmented epithelium

**SCFA**, short‐chain fatty acid

**SFA**, saturated fatty acid

**TBC**, tubulobulbar complex

**VLCFA**, very long‐chain fatty acid

Fatty acids (FAs) have a wide range of physiological roles as an energy source, membrane phospholipid component, donor substrate for protein acylation, and lipid mediators (or precursor) 1, 2, 3. FAs can be classified based on several structural features. From their carbon chain lengths, FAs are defined as short‐chain FAs (SCFAs; fewer than six carbons), medium chain FAs (MCFAs; from 6 to 12 carbons), long‐chain FAs (LCFAs; from 14 to 20 carbon chains), and very long‐chain FAs (VLCFAs; over 22 carbon chains). FAs are also classified by double bond number as saturated FAs (SFAs; no double bonds), monounsaturated FAs (MUFAs; one double bond), and polyunsaturated FAs (PUFAs; two or more double bonds). Unsaturated FAs are also classified by the position of the first double bond from their methyl– (ω–) end and are commonly designated as being ω‐9, ω‐6, or ω‐3 FAs 4.

Omega‐3 FAs, including alpha‐linolenic acid (ALA), eicosapentaenoic acid (EPA), and docosahexaenoic acid (DHA), are essential nutrients for normal development and health 5. Mammals cannot synthesize ω‐3 FA *de novo* because they lack the enzymes to produce double bonds at the ω‐3 position and therefore must obtain ω‐3 FAs from diet 6. ω‐3 FAs are initially formed in the chloroplasts of green plants, algae, and plankton, and they are concentrated and reach relatively high levels in fish and seafoods. Health benefits of dietary ω‐3 FA intake attracted much interest by a study in 1978 which demonstrated a low incidence of acute myocardial infarction in Greenland Eskimos who have diets very rich in ω‐3 FAs 7. To date, numerous studies have demonstrated the importance of ω‐3 FAs in various tissues including heart, brain, retina, and testes 8, 9, 10, 11.

The biological roles of ω‐3 FAs consist of four distinct categories: lipid mediator precursor, transcriptional regulators, modulator of membrane protein functions, and component of glycerophospholipids (GPLs). As lipid mediators, it is reported that EPA‐ and DHA‐derived lipid mediators are less potent to stimulate platelet aggregation than arachidonic acid (AA)‐derived lipid mediators, which they displace 12, 13, and more recent studies revealed that EPA and DHA are also converted to proresolving lipid mediators, such as protectins, resolvins, and maresins 14, 15. As transcriptional regulators, EPA and DHA act as ligands for several nuclear receptors/transcriptional factors, including peroxisome proliferator‐activated receptor alpha (PPARα) 16. By binding PPARα, EPA and DHA reduce circulating triglyceride levels, and EPA and DHA supplements are clinically used to treat hyperlipidemia 16. As modulators of membrane proteins, ω‐3 FAs are reported to directly bind to several ion channels and thereby modulate their activities 17, 18, 19. This activity occurs in myocardium and may be important for some of the cardioprotective effects associated with fish oil consumption. In addition to these effects, since ω‐3 FAs, especially DHA, are highly flexible, their incorporation into membrane GPLs affects membrane physicochemical properties 20, 21. However, compared to the other biological roles of PUFAs, the mechanisms of incorporation and significance of DHA in membranes have remained obscure. In recent years, studies utilizing gene knockout strategies and molecular dynamics simulations have begun to clarify the importance of ω‐3 PUFA incorporated into membrane GPLs as well as the cellular mechanisms by which DHA incorporation occurs. Here, we review recent progress to understand the roles of ω‐3 PUFAs, especially DHA, in membrane GPLs.

## Production of ω‐3 PUFA‐containing GPLs

Cellular membranes are composed of several types of GPLs which are classified by their polar head groups, such as phosphatidic acid (PA), phosphatidylcholine (PC), phosphatidylethanolamine (PE), phosphatidylserine, phosphatidylglycerol, and phosphatidylinositol 22. GPLs are also classified by their FA linkages to the hydroxyl group of the glycerol backbone as diacyl type, alkyl‐acyl type, and alkenyl‐acyl type (plasmalogens) 23. Due to the many combinations of FAs at *sn*‐1 and *sn*‐2 positions combined with the variation in linkages and classes, cellular membranes may contain over 1000 distinct species of GPLs 24.

**Figure 1:**
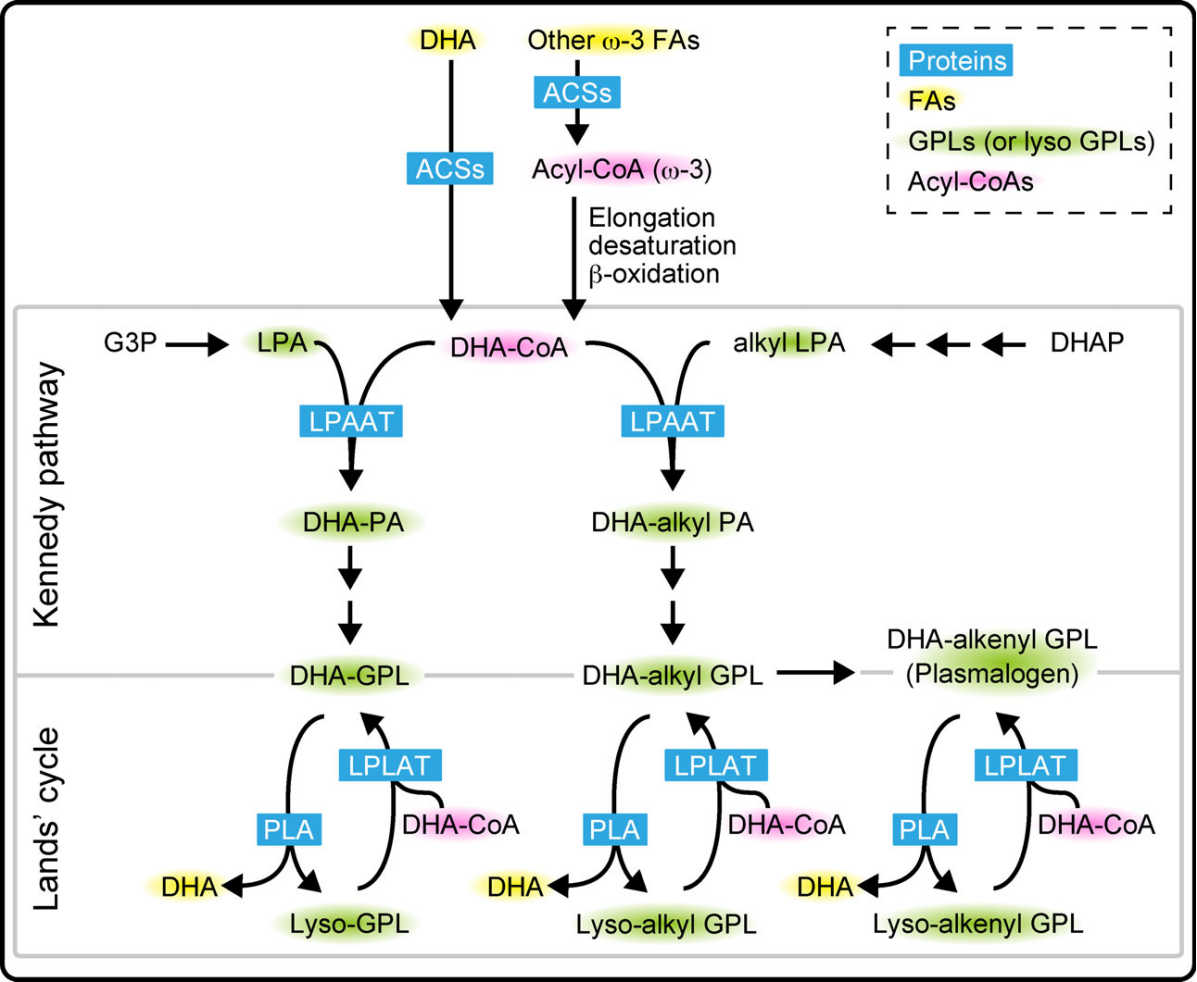
The synthetic pathways of DHA‐containing GPLs (DHA‐GPLs) in mammalian cells. Since mammalian cells cannot synthesize ω‐3 FAs, dietary uptake of DHA or other ω‐3 FAs such as ALA is required to generate DHA‐containing GPLs. In the cells, DHA‐containing GPLs are formed by two independent pathways, the *de novo* pathway (Kennedy pathway) and the remodeling pathway (Lands’ cycle). In the Kennedy pathway, DHA is mainly incorporated into the *sn*‐2 position of PA by the action of LPAATs. PA is a common intermediate for all GPL synthesis, and DHA‐containing PA is converted to other DHA‐containing GPLs. Alternatively, DHA may be incorporated into GPLs by the concerted actions of PLAs and LPLATs in the Lands’ cycle. DHAP, dihydroxyacetone phosphate.

Diversity of FA compositions in membrane GPLs is mainly generated by the action of lysophospholipid acyltransferases (LPLATs) and occurs through two different pathways: the *de novo* pathway (Kennedy pathway) and the remodeling pathway (Lands’ cycle; Fig. 1) 25. Glycerol‐3‐phosphate (G3P) acyltransferases and lysophosphatidic acid acyltransferases (LPAATs) function in the Kennedy pathway. LPAATs, which use lysophosphatidic acid as an acyl acceptor, regulate FA compositions of GPLs during production of PA, a common intermediate for GPLs and neutral lipid synthesis. In the Lands’ cycle, phospholipase (PLA)‐ and LPLAT‐mediated deacylation–reacylation reactions ensure remodeling of membranes and contribute to diversity of cellular GPLs. The coordinated actions of both the Kennedy pathway and the Lands’ cycle are required to generate the full diversity of FA compositions of membrane GPLs. These compositions differ among tissues and cell types, and may be further modified to in response to physiological cues. In the regulation of AA level in GPLs, FA remodeling by LPCAT3 regulates levels of AA‐containing GPLs and is required for triglyceride transport in liver and small intestine 26, 27, 28. It is suggested that AA incorporation is dependent on both the Kennedy pathway and Lands’ cycle 29. In contrast, DHA incorporation into GPLs may occur primarily in the Kennedy pathway, because it is reported that cytidine diphosphate‐ethanolamine:diacylglycerol (DAG) ethanolaminephosphotransferase, which converts DAG to PE, and PE *N*‐methyltransferase, which converts PE to PC, prefer DHA‐containing species as substrates 30, 31. In addition, a recent study comparing *in vitro* LPLAT activities and tissue PC compositions also suggested that levels of DHA‐containing GPLs may be primarily regulated by LPAAT activities functioning in the Kennedy pathway rather than other LPLATs functioning in the Lands’ cycle 29. Recently, we also reported that in LPAAT3 KO mice DHA‐containing GPLs were greatly decreased 32, 33, consistent with the idea that DHA‐containing GPLs are primarily produced through the Kennedy pathway.

Lysophospholipid acyltransferases require acyl‐CoA as donors in their acylation reactions, therefore acyl donor substrate supply is also an important determinant of FA compositions of cellular GPLs. In cells, FAs are activated to acyl‐CoA by acyl‐CoA synthetases (ACSs) 34 and are subsequently either used as substrates by LPLATs or else further elongated and/or desaturated by the actions of FA elongases and desaturases 22, 35. Alternatively, acyl‐CoAs are subjected to β‐oxidation when cells require energy 36, 37. Although mammals cannot synthesize ω‐3 FAs *de novo*, DHA and EPA can be formed from dietary α‐linolenic acid (ω‐3, C18:3) through FA elongation, desaturation, and beta‐oxidation steps 38. Fatty acid desaturases (FADS1 and FADS2) and elongation of very long‐chain fatty acid enzymes (ELOVL2 and ELOVL4) are required for this formation of DHA‐ and EPA‐CoA 39. Genetic deletion of ELOVL2 in mice leads to the dramatic reduction of DHA in hepatic GPLs and triglycerides 40, indicating that DHA formed from precursor ω‐3 FAs provides an important source of donor substrate for acylation reactions and also affects the diversity of FA compositions in membrane GPLs.

## Effects of GPL compositions on the function of cellular membrane

Glycerophospholipids are major components of biological membranes, and their FA compositions may profoundly affect cellular processes. Based on their physiochemical properties, PUFAs and especially DHA are predicted to impart unique characteristics to biological membranes.

### Membrane fluidity

**Figure 2:**
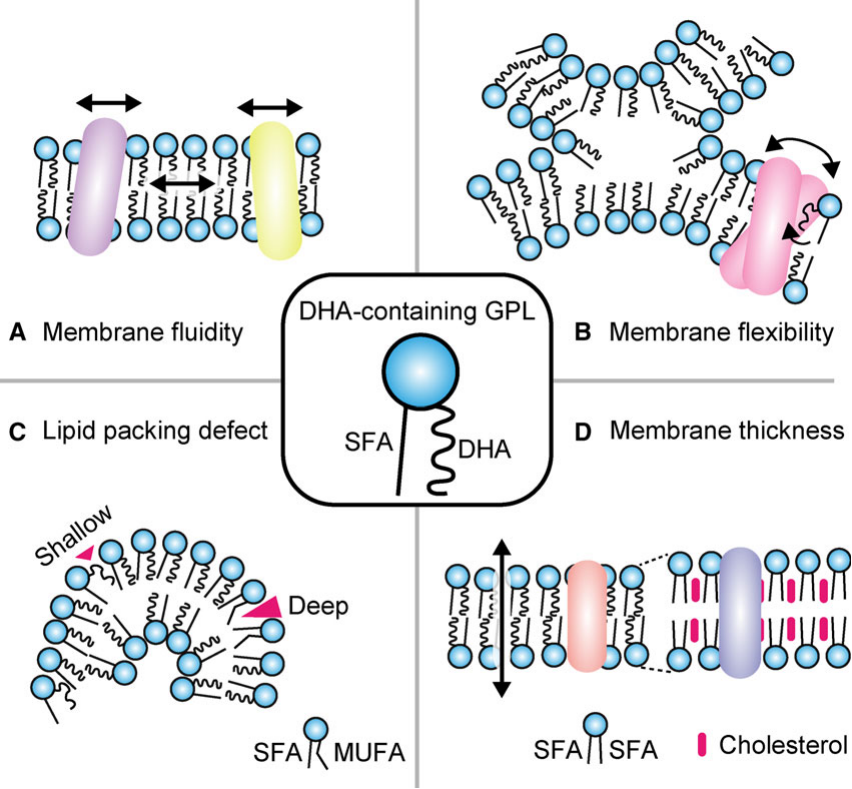
The roles of DHA‐containing GPLs in membranes. (A) Enrichment of PUFA, including DHA, in membrane GPLs may impart fluidity to membranes. Highly fluid cellular membrane may enhance membrane dynamics to facilitate processes such as lateral diffusion of membrane proteins. (B) Highly flexible and DHA‐containing GPLs facilitate the fusion and fission of membranes. Highly deformable DHA‐rich membranes also support rapid conformational changes of membrane proteins. (C) Strong curvature of membrane leads to partial exposure of hydrophobic tails of GPLs to the cytosolic surface, a phenomenon termed lipid packing defect. DHA‐containing GPLs may promote formation of more shallow defects than MUFA‐containing GPLs, and this depth of lipid packing defect may affect the recruitment of several lipid packing defect‐sensing proteins. (D) Lipid bilayers composed of DHA‐containing GPLs are thinner compared to those composed of disaturated phospholipids and cholesterol. Loosely packed thin membranes may have increased permeability to ions and small molecules. Membrane thickness is also a determinant of membrane protein localization and activity.

Compared to SFAs, the structure of unsaturated FAs tends to be more curved due to that *cis* double bonds make a ‘kicked’ structure 41. Since this curvature decreases the molecular interaction between FA chains of neighboring GPLs, enrichment of PUFA‐containing GPLs increases membrane fluidity and affects many cellular functions that are dependent on membrane dynamics (Fig. 2A) 41, 42.

### Membrane flexibility

In addition to the effect on membrane fluidity, molecular dynamics simulations reveal a lowered barrier for rotation about vinyl‐methylene bonds of FAs imparts an increased propensity for flexibility to PUFAs, especially DHA, over SFAs or MUFAs 20, 21, and DHA incorporated into GPLs accordingly affects membrane flexibility (Fig. 2B) 43. Indeed, high DHA incorporation is reported to facilitate membrane deformation in processes such as membrane fusion and fission 44. Polar head groups of GPLs are also known to affect membrane fusion and fission. As opposed to cylinder‐shaped GPLs with large polar head groups (e.g., PC), cone‐shaped GPLs with small polar head groups (e.g., PA and PE) form inverted hexagonal‐II phases and are reported to promote membrane fusion and fission 45. In this regard, enrichment of DHA in PE rather than in PC might be important for affecting cellular functions involving membrane fusion and fission in several tissues. Additionally, highly flexible DHA‐containing membranes may influence or promote conformational changes of membrane proteins (Fig. 2B) 46.

### Lipid packing defects

In highly curved membrane, as occurs during membrane fission, the hydrophobic FA tails of GPLs are partially extruded from the hydrophobic interior of the bilayer and physically exposed on the hydrophilic surface. This phenomenon is termed ‘lipid packing defect’ (Fig. 2C) 47. Several proteins are known to sense and bind lipid packing defects via hydrophobic domains or amphipathic helices. FA compositions of membranes also affect generation and biophysical properties of lipid packing defects, and high PUFA content is suggested to favor generation of more shallow defects. Depending on the molecular interactions between these defects and the defect‐sensing motifs, it is possible that the sensing proteins may detect and respond differently to the packing defects depending on the PUFA content of very highly curved membranes (Fig. 2C) 48.

### Membrane thickness

Fatty acid chain length and *cis* double bonds of GPLs are known to affect lipid bilayer thickness (Fig. 2D) 49, 50. Liposome lipid bilayers composed of PUFA‐containing GPLs are thinner than those composed of SFAs, and it is thought that DHA‐enriched membrane may be thin, as has been reported for membranes of rod photoreceptor disks, which have high amounts of DHA‐containing GPLs and are very thin 51. Since transmembrane domain lengths of integral membrane proteins have correlations with membrane thickness, enrichment of DHA‐containing GPLs may affect the localization and trafficking of membrane proteins 52. Also, thinner and loosely packed DHA‐enriched membranes are reported to have increased permeability to small polar molecules (Fig. 2D) 53. This is also an important property of DHA in cellular membranes.

## Roles of DHA‐containing GPLs in various tissues

Fatty acid compositions of GPLs vary among tissues and cell types, suggesting the compositions are tightly regulated and impart specific biochemical properties to membranes based upon physiological requirements. DHA‐containing GPLs are abundant in retina, testes, brain, heart, and skeletal muscle and may have important functions in each of these tissues 8, 9, 10, 29, 54.

### The roles of DHA‐containing GPLs in the retina

**Figure 3:**
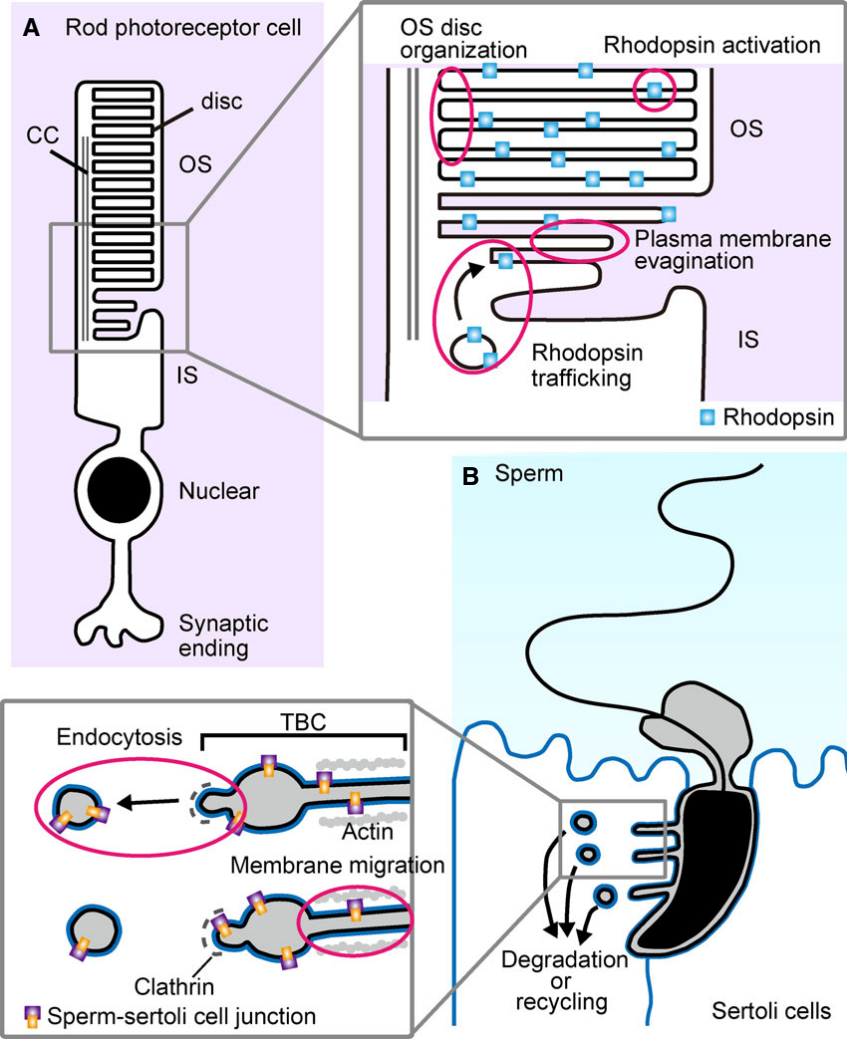
The proposed functions of DHA‐containing GPLs in retinal rod photoreceptor cells (A) and mature sperm (B). (A) DHA‐containing GPLs are enriched in OS disks. OS disks are formed by plasma membrane evagination, and DHA‐rich flexible membranes may be critical for this process. Highly flexible DHA‐rich membranes might also support rapid conformational changes of rhodopsin after light stimulation. The phenotypes of Mfsd2a‐ and LPAAT3‐deficient mice suggest that DHA‐containing GPLs are required for the OS disk organization and maintenance, as well as rhodopsin trafficking. CC, connecting cilium. (B) Defective spermatogenesis in LPAAT3 KO mice indicates the critical role of DHA‐containing GPLs in the final step of sperm formation. In this step, excess sperm cytoplasm and plasma membranes are removed with sperm–Sertoli cell junctions through apical TBC. LPAAT3 KO sperm possess an excess of cytoplasm and Sertoli cell membranes around the sperm head, suggesting that DHA‐containing GPLs promote clathrin‐mediated endocytosis and/or rapid membrane migration through the actin‐lined narrow tubules.

It has long been known that retinal membrane contains a high amount of DHA 55. In the retina, DHA‐containing GPLs are abundant in outer segment (OS) disks of rod photoreceptor cells 33, 56. OS disk is a unique organelle specialized in phototransduction. Rhodopsin, a dim light‐sensitive G protein‐coupled receptor, is a major protein component (> 90%) of rod photoreceptor OS disks 57. The interaction between rhodopsin and DHA in GPL was observed by several approaches including molecular dynamics simulations and nuclear magnetic resonance studies 58, 59 Those studies suggest that the flexible property of DHA permits very rapid conformational changes of rhodopsin after photoactivation in the membrane (Fig. 3A).

Since photoreceptors are segregated from blood flow by blood–retinal barrier (BRB) composed of retinal capillary endothelial cells and retinal pigmented epithelium (RPE) 60, it has been a long‐standing mystery how photoreceptors obtain such a high amount of DHA. Recent studies identified two important molecules involved in the transport of DHA from circulation into the retina. One is adiponectin receptor 1 (AdipoR1) 61 and the other is major facilitator superfamily domain‐containing protein 2a (Mfsd2a) 62. AdipoR1 is a receptor for adiponectin, an adipocyte‐derived hormone implicated in insulin sensitivity 63. Besides the well‐studied function of adiponectin in metabolic tissues, Rice *et al*. 61 reported the expression and function of AdipoR1 in the retina. AdipoR1 KO mice showed progressive retinal degeneration with a dramatic reduction of unesterified DHA, DHA‐containing GPLs, and very long‐chain PUFA‐containing GPLs in the retina. Surprisingly, adiponectin KO mice did not show retinal degeneration, indicating the functions of AdipoR1 in retina may be independent of cognate ligand binding. AdipoR1 is expressed both in RPE and photoreceptor cells, therefore AdipoR1 may be involved in trafficking of DHA from circulation into RPE as well as from RPE into photoreceptor cells. Rice *et al*. 61 also reported AdipoR1 overexpression‐enhanced DHA incorporation into human spontaneously transformed RPE cells, and an association reported for a single nucleotide polymorphism on the AdipoR1 locus and age‐related macular degeneration in humans further supports an important role for AdipoR1 in human vision 64. In addition to AdipoR1, Mfsd2a, which is highly expressed in RPE, was also shown to be involved in DHA incorporation across BRB. DHA‐containing GPLs in Mfsd2a KO mouse retina were reduced by half compared to wild‐type, resulting in OS disorganization and rhodopsin mislocalization 62. Unlike AdipoR1, Mfsd2a transports DHA as DHA‐containing lysophosphatidylcholine (LPC) rather than unesterified DHA in a sodium‐dependent manner 65. Taken together, these studies indicate retinas may obtain DHA by at least two independent mechanisms. It is intriguing that although DHA‐containing GPLs are decreased in both AdipoR1‐ and Mfsd2a‐KO mice, dramatic decreases of VLCFA species occur only in AdipoR1 KO mice 61, 62. These results suggest that the uptake mechanism and form of the DHA may also affect its incorporation into GPLs and metabolism. Very recently, novel VLCFA‐derived neuroprotective lipid mediators, termed ‘elovanoids’, have been reported 66. These VLCFA‐derived lipid mediators may contribute to the phenotypic differences between AdipoR1‐ and Mfsd2a‐KO mice.

The enzyme responsible for DHA incorporation into retinal GPLs was reported recently by our group. LPAAT3 KO mice showed a specific and almost complete lack of DHA‐containing GPLs in the retina, while AA‐containing GPLs were dramatically increased 33. LPAAT3 KO mice showed retinal degeneration with abnormal morphology/organization of photoreceptor OS disks, indicating the critical requirement of DHA in retina and suggesting that increased AA‐containing GPLs cannot rescue the functions of DHA‐containing species. Abnormal disk morphology/disorganization in Mfsd2a‐ and LPAAT3‐KO mice raise the possibility that enrichment of DHA‐containing GPLs is required not only for rhodopsin activation but also photoreceptor OS disk organization (Fig. 3A). Recent studies clearly showed that nascent rod photoreceptor OS disks are formed by evagination of plasma membrane at a neck region of OS (Fig. 3A) 67, 68, 69. OS disks are thin, and DHA‐containing GPLs may impart flexibility to the membranes that contributes to formation of strong curvatures at the edge of disk membranes in photoreceptor cells. Mislocalization of rhodopsin in the perinuclear region was seen in both Mfsd2a and LPAAT3 KO mice (Fig. 3A) 33, 62. As DHA‐containing GPLs may also promote membrane fusion and fission, another possible function for DHA‐containing GPLs in photoreceptor cells might be to promote trafficking of rhodopsin from endoplasmic reticulum to the plasma membrane via the Golgi apparatus (Fig. 3A) 70.

### The roles of DHA‐containing GPLs in the testes

The testes are also known to possess high amounts of DHA in their membranes, especially in mature spermatids 9, 71. The importance of PUFAs in the testes was demonstrated using FADS2 KO mice by two independent groups 72, 73. FADS2 KO mice, deficient in highly unsaturated FAs (possessing four or more double bonds), showed multiple abnormalities including male infertility. FADS2 KO mice lacked elongated spermatids due to a failure in acrosome formation, a process essential for maturation of round to elongating spermatids 72, 73, 74. Acrosome formation requires two membrane‐associated steps where highly fluid and flexible membrane may be required: the budding and fission of acrosomal vesicles from Golgi apparatus, and the fusion of acrosomal vesicles 75. A subsequent study further demonstrated that the defect of spermatid maturation in FADS2 KO mice could be completely rescued by dietary DHA supplementation, while rescue by AA supplementation was partial 10. These studies indicate an essential role of DHA in spermatid maturation.

Why is DHA, but not AA, essential for the spermatid maturation? Further evidence for the specific requirement of DHA in spermatid maturation has been demonstrated using LPAAT3 KO mice. LPAAT3 was originally identified as an LPAAT enzyme highly expressed in the testes which showed preference to utilize PUFA‐CoA in *in vitro* assays 76. LPAAT3 expression increased in testes during sexual maturation, suggesting a role in male reproduction. Male LPAAT3 KO mice showed complete infertility due to abnormal sperm formation 32. Similar to the GPL compositions in LPAAT3 KO retinas, DHA‐containing GPLs in LPAAT3 KO testes and isolated testicular mature spermatids were dramatically decreased, while AA‐containing GPLs were compensatorily increased. In contrast to FADS2 KO mouse spermatids, LPAAT3 KO spermatids are able to mature into elongated spermatids and form acrosomes, suggesting that in these processes other PUFA‐containing species may compensate for the lack of DHA‐containing species. However, LPAAT3 KO mice failed to form fully mature fertile spermatids due to a defect in elimination of excess cytoplasmic component. At the final step of spermatogenesis, surrounding Sertoli cells uptake excess spermatid cytoplasm and plasma membrane along with spermatid–Sertoli cell junctions via tubulobulbar complexes (TBCs) and release fully developed spermatids into seminiferous tubules (Fig. 3B) 77. TBCs are composed of actin‐lined narrow (~ 50 nm in diameter) tubules, endoplasmic reticulum‐surrounded bulbous portions, and clathrin‐coated pits (Fig. 3B). At the tips of TBCs, spermatid and Sertoli cell membranes along with spermatid cytoplasm are internalized by Sertoli cells via clathrin‐mediated endocytosis and degraded by lysosomes‐dependent mechanisms. The defect in spermatogenesis in LPAAT3 KO mice suggests that DHA‐containing GPLs may impart the required flexibility to sperm membranes necessary for efficient endocytosis through TBC tubules, and substitution of other PUFAs for DHA is not able to provide the membrane properties required for this process. In this regard, the mechanisms underlying abnormal photoreceptor disk morphology and spermatid maturation in LPAAT3 KO mice might be similar. Together with the fact that cone‐shaped GPLs such as PE facilitate membrane fusion and fission processes 45, 78, the higher proportion of DHA‐containing species in PE (~ 40% in total PE) than in PC (~ 10% in total PC) in elongated spermatids may also be important for their maturation.

Sperm undergo further maturation during passing through the epididymis. In this process, sperm membrane is reported to be more enriched in PUFAs including DHA 79, suggesting DHA‐containing GPLs are also important for the sperm functions in addition to their required role in spermatogenesis. It is reported that genetic deletion of group III secreted PLA_2_ (sPLA_2_‐III), which is highly expressed in caput epididymis, caused decreased DHA‐ and DPA‐containing GPLs of sperm in cauda epididymis, but not in caput epididymis 80. Since the sPLA_2_‐III KO sperm have defects in fertility, enrichment of DHA‐ and DPA‐containing GPLs during passage through the epididymis is also required for the sperm maturation.

### The roles of DHA‐containing GPLs in the brain

The brain is also abundant in DHA‐containing GPLs 8. DHA has been linked to brain development, functions, and diseases 81, 82. DHA may be preferentially supplied into the brain through the blood–brain barrier (BBB) as DHA‐containing LPC 83, 84. In support of this, loss of Mfsd2a, which is expressed in endothelial cells of the BBB, resulted in a dramatic reduction of DHA‐containing GPLs in the brain 65. This suggests that both brain and retina depend upon the Mfsd2a‐mediated transport of DHA‐containing LPC to incorporate DHA through BBB and BRB, respectively. Mfsd2a KO mice showed brain dysfunctions, such as cognitive deficits, severe anxiety, and microcephaly, suggesting the importance of DHA in brain 65, and homozygous inactivating mutations in Mfsd2a have been likewise found in patients with severe microcephaly 85, 86. In addition to the decrease of DHA‐containing GPLs in Mfsd2a KO mouse brain, it is also reported that BBB of these mice are leaky 87. A recent study revealed that Mfsd2a‐mediated DHA incorporation controls the BBB permeability by suppressing the caveolae‐mediated transcytosis 88. It is important that Mfsd2a and Caveolin‐1 double KO could rescue the leaky BBB but not microcephaly phenotype in Mfsd2a KO mice 88. This suggests that BBB leakage is not related to the microcephaly in Mfsd2a KO mice. Therefore, further studies are required to clarify the molecular mechanisms underlying the behavioral abnormalities and microcephaly in Mfsd2a KO mice. Recently, fatty acid‐binding protein 5 (FABP5) was also reported to incorporate DHA into the brain through BBB but in unesterified form 89. Although the DHA incorporation into BBB is reduced in FABP5 KO mice, the contribution of FABP5 to supply DHA across the BBB may be minor compared to Mfsd2a.

A variety of effects of DHA in the brain, such as enhanced neurotransmission, neuronal outgrowth, and synaptic plasticity, have been demonstrated using various approaches 90. These effects may be exerted, at least in part, by altering membrane physical properties that affect fusion/fission and membrane‐involved signal transduction processes. However, the precise mechanisms by which DHA‐containing GPLs contribute to brain functions are largely unknown. An imaging mass spectrometric study demonstrated that PUFA‐containing GPLs are higher in axon tips than cell bodies of cultured neurons 91. This suggests that PUFA‐containing GPLs may have a role in effective release of neurotransmitters via impacting membrane physical properties.

Decreased DHA levels in the brain are reported under several conditions including aging and Alzheimer's disease 92. A biochemical study utilizing liposomes suggesting that gamma‐secretase produced the more pathologic amyloid‐beta_42/43_ (Aβ_42/43_) from amyloid precursor protein in thicker liposomes, containing longer acyl chains, than in thinner liposomes 93, 94, 95. This observation suggests that DHA‐containing thin membranes may inhibit production of Aβ_42/43_ and the onset of Alzheimer's disease. However, the effects of DHA on Alzheimer's disease and cognitive functions differs among the studies and remains controversial 96, 97.

### The roles of DHA‐containing GPLs in heart

Clinical and epidemiological studies support that ω‐3 FA consumption promotes cardiovascular health and may prevent or improve coronary heart disease 11. The precise roles of ω‐3 FAs in cardiac physiology are not well understood, and ω‐3 FA consumption may affect cardiac functions by both indirect and direct mechanisms. Indirect mechanisms include effects on plasma lipid profiles, the vascular system, autonomic functions, and whole‐body metabolism, while several direct effects of fish oil may be mediated by ω‐3 FAs that are incorporated into myocardial membranes. These direct effects may include improved heart rate and resistance to ischemic stress, arrhythmia, and congestive heart failure 98.

Elevated resting heart rate is a major risk factor for cardiovascular mortality, and regular ω‐3 supplementation lowers intrinsic heart rate and protects against arrhythmias. ω‐3 FAs might alter heart rate by several potential mechanisms, and studies of heart transplant patients 99, isolated rat hearts 100, and isolated rabbit pacemaker cells 101 indicate a direct effect independent of central autonomic functions 98, 102. Although the physiological functions of ω‐3 FAs within the myocardial membranes are not clear, GPL‐incorporated ω‐3 FAs might alter membrane fluidity, permeability, or electrophysiological properties and thereby modulate ion channel functions and impact heart rate; similar mechanisms might also provide protection from Ca^2+^ overload and irregular cytosolic Ca^2+^ fluctuations, causing antiarrhythmic effects 102.

Although both DHA and EPA are abundant in fish oil, they may be differentially incorporated into myocardial GPLs and also differ in their effects on cardiac physiology 98. In rats, levels of DHA are higher than EPA in myocardial membrane GPLs, and following fish oil supplementation DHA is preferentially incorporated into myocardial phospholipids over EPA regardless of DHA and EPA levels in the supplement 103. In humans, DHA‐containing GPLs were higher than EPA‐containing species in right atrial appendages removed during bypass surgery 104, and DHA‐containing GPL was preferentially increased by preoperative dietary fish oil supplementation 105. Functional outcomes between supplementation with DHA or EPA may also differ. DHA but not EPA supplementation was efficacious to lower heart rate in humans 106, 107. In spontaneous hypertensive rats, DHA but not EPA supplementation inhibited ischemia‐induced arrhythmia 108. Further studies are required to uncover the cellular mechanisms underlying the selective incorporation and protective effects of DHA in myocardial membranes.

### The roles of DHA‐containing GPLs in skeletal muscles

Skeletal muscle undergoes metabolic adaptation in response to physiological cues such as physical activity and nutritional status. Adaptation of skeletal muscle to regimes of endurance exercise training includes a switching of glycolytic to more oxidative fiber types and is associated with whole‐body metabolic improvements such as increased endurance and resistance to obesity 109. The adaption to endurance exercise is also associated with changes in the FA compositions of GPLs, and several lines of evidence support a role for increased incorporation of DHA into GPLs.

In several studies in mice, rats, and humans, DHA‐containing GPLs were increased by endurance exercise training independently of diet, and DHA‐containing GPL levels showed positive correlations with oxidative capacity of the skeletal muscle 110, 111, 112, 113, 114. In mice, exercise‐induced increases of DHA in PE were observed in extensor digitorum longus muscles, whereas more oxidative soleus muscle already had high DHA in PE even without training 110. In rats, DHA‐containing PE content was higher in oxidative than in glycolytic vastus lateralis 114. In humans, DHA‐containing GPL levels were higher in vastus lateralis muscles of endurance‐trained young men and correlated with increased type I oxidative fiber percentage 113. In another study in humans, 4 weeks of one‐leg exercise training led to increased DHA‐containing GPLs as well as increased citrate synthase activity compared to the untrained leg 112.

Reports also indicate that dietary DHA supplementation may promote endurance even without training. In untrained rats, 9 weeks of dietary DHA supplementation resulted in increased skeletal muscle DHA‐containing GPL levels as well as increased capacity for endurance exercise. The DHA supplementation altered several metabolic parameters in isolated and permeabilized skeletal muscle myofibers, indicating improved mitochondrial functions 115. Also in birds, 6 weeks of DHA or EPA supplementation strongly increased oxidative enzyme activities in flight muscles of confined quail concurrent with enhanced DHA‐ or EPA‐containing GPL contents 116.

The mechanisms and functions of enhanced DHA‐containing GPL in endurance‐trained skeletal muscle require further elucidation. PPARγ coactivator‐1 α (PGC1α) is activated by exercise downstream of adenosine monophosphate‐activated protein kinase (AMPK) and is a key regulator of mitochondrial biogenesis and cellular metabolism. In one study, mice overexpressing PGC1α in skeletal muscle had enhanced levels of several DHA‐containing GPL species 110. DHA incorporation into the same GPL species was also increased by endurance training in a PGC1α‐dependent manner, indicating that training‐induced increases in DHA‐containing GPLs may be transcriptionally regulated downstream of exercise‐induced signaling pathways. The functional significance of the enhanced DHA‐containing GPLs is not clear but may affect cellular metabolism through several mechanisms. Increased DHA content incorporation into membrane GPLs may alter biophysical properties of the membranes to affect cellular bioenergetics. In support of this theory, fish oil supplementation to active men resulted in increased DHA and EPA in skeletal muscle mitochondrial GPLs that was accompanied by improvements in several mitochondrial respiratory parameters including adenosine diphosphate (ADP) sensitivity 117. Enhanced DHA content in membrane GPLs may also affect levels of free DHA or its metabolites which are known ligands for PPAR nuclear receptors and may alter their activities to transcriptionally regulate metabolic gene expressions 118, 119, 120. Further identification of the enzymes and mechanisms that regulate DHA‐containing GPL levels in skeletal muscle is required to clarify many of the metabolic functions of DHA.

## Future perspectives and conclusion remarks

Omega‐3 PUFAs have important functions on human health, and recent studies have clarified some of the molecular mechanisms underlying the effects of DHA in various tissues. Several important molecules involved in the incorporation of DHA into cells and production of DHA‐containing GPLs have been identified, including LPAAT3, Mfsd2a, and AdipoR1 32, 33, 61, 65. However, very basic questions still remain as to how DHA is incorporation is regulated and the precise functions of DHA in membrane GPLs.

The studies using LPAAT3 KO mice demonstrated the essential role of LPAAT3 to generate DHA‐containing GPLs in retinas and testes 32, 33. As LPAAT3 is not ubiquitously expressed and other enzymes such as LPAAT4 may be involved in DHA‐containing GPL production 121, further studies are required for a comprehensive understanding of how DHA‐containing GPLs are generated and metabolized in the body. It is possible that FA remodeling in the Lands’ cycle is also important in the production of DHA‐containing GPLs in various tissues. It is intriguing that Mfsd2a‐mediated DHA incorporation is important into brain and retina but not liver and skeletal muscle 62, 65. This suggests that Mfsd2a is specifically required to incorporate DHA into GPL in areas isolated from blood flow. However, a dramatic induction of Mfsd2a mRNA expression during cold exposure and fasting in brown adipose tissue and liver is also reported 122, 123, suggesting these tissues may also use the DHA‐containing LPC as a source of DHA under specific situations. It is unknown why these tissues obtain DHA as LPC in a sodium‐dependent manner. The production mechanisms of DHA‐containing LPC and its sources also remain open questions.

Although DHA in GPLs may have a specific role in various cellular processes by imparting membrane flexibility, it is also easily oxidized under oxidative stress and generates toxic lipid peroxides 124. Lipid peroxidation is known to cause apoptosis 125, and recent studies have reported a novel iron‐dependent cell death, termed ‘ferroptosis’, that is also caused by lipid peroxidation 126. Thus, cells with PUFA‐enriched membranes may require protective mechanisms against various oxidative stresses. In this regard, it is proposed that PUFA incorporation into plasmalogens may be important because the vinyl double bond linkage is preferentially oxidized, preventing oxidation of the PUFA and possibly mitigating cellular lipid peroxidation reactions 127. Further studies are required to understand the cellular mechanisms that protect PUFA‐enriched membranes from oxidative stresses.

In addition to understanding their biological effects and functions, how cells are able to distribute and regulate PUFA‐enriched membranes also needs to be addressed. In the case of neurons, accumulation of PUFA‐containing PC species in axon tips is actin dependent, suggesting PUFA‐containing GPL‐enriched vesicles may be selectively sensed and trafficked 111. Incorporated PUFAs are also freed from the membranes, and GPL‐esterified DHA is not only a membrane structural component but also a potential precursor for lipid mediators and other metabolites. Comprehensive understanding of how DHA‐enriched membranes are generated, trafficked, and metabolized will help clarify biological functions and patho‐physiological roles of DHA.
